# Computational Drug Screening Identifies Compounds Targeting Renal Age-associated Molecular Profiles

**DOI:** 10.1016/j.csbj.2019.06.019

**Published:** 2019-06-25

**Authors:** Christian Koppelstaetter, Johannes Leierer, Michael Rudnicki, Julia Kerschbaum, Andreas Kronbichler, Anette Melk, Gert Mayer, Paul Perco

**Affiliations:** a Department of Internal Medicine IV (Nephrology and Hypertension), Medical University Innsbruck, Innsbruck, Austria; b Department of Kidney, Liver and Metabolic Diseases, Children's Hospital, Hannover Medical School, Hannover, Germany

**Keywords:** Renal aging, Chronic kidney disease progression, Computational compound screening, Drug repurposing, Anti-aging

## Abstract

Aging is a major driver for chronic kidney disease (CKD) and the counterbalancing of aging processes holds promise to positively impact disease development and progression.

In this study we generated a signature of renal age-associated genes (RAAGs) based on six different data sources including transcriptomics data as well as data extracted from scientific literature and dedicated databases. Protein abundance in renal tissue of the 634 identified RAAGs was studied next to the analysis of affected molecular pathways. RAAG expression profiles were furthermore analysed in a cohort of 63 CKD patients with available follow-up data to determine association with CKD progression. 23 RAAGs were identified showing concordant regulation in renal aging and CKD progression. This set was used as input to computationally screen for compounds with the potential of reversing the RAAG/CKD signature on the transcriptional level. Among the top-ranked drugs we identified atorvastatin, captopril, valsartan, and rosiglitazone, which are widely used in clinical practice for the treatment of patients with renal and cardiovascular diseases. Their positive impact on the RAAG/CKD signature could be validated in an in-vitro model of renal aging.

In summary, we have (i) consolidated a set of RAAGs, (ii) determined a subset of RAAGs with concordant regulation in CKD progression, and (iii) identified a set of compounds capable of reversing the proposed RAAG/CKD signature.

## Graphical abstract

**Figure:**
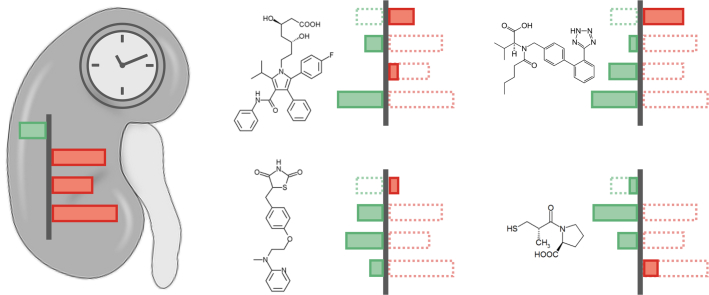

## 1 Introduction

Global life expectancy increased over the last 35 years from 62 to 72 years, while in the same time period causes of death and the profile of diseases significantly changed [1]. More individuals reach higher age and develop chronic diseases. Among them, kidney diseases are still a rising cause of disability adjusted life years and amid the thirty leading causes of death. Age in this context is a heterogeneous term that needs to be differentiated into chronological age defined as counting time in days, months, and years from birth, and biological age as a functional decline of cellular function leading to alterations of organs and eventually the whole organism [2]. Various triggers have been identified that can cause or accelerate the process of aging. Scientific effort has been invested to understand these mechanisms and to find ways to decelerate the aging process [[3], [4], [5]].

The process of renal aging leads to mass loss of renal cortex as well as to loss of function including vascular abnormalities, reduced renal plasma flow and a decline in glomerular filtration rate (GFR) with a mean loss of GFR of 0.75 mL/min/1.73 m^2^ per year. Histologically, renal aging manifests in interstitial fibrosis, glomerulosclerosis, tubular atrophy and thickening of the intima of arteries [6].

Several causative mechanisms involved in renal aging have been catalogued by Halloran and Melk in 2001 for the first time [7]. Cellular senescence triggers many of these pathological changes and senescence as well as aging promote and influence each other [8]. Two major pathways were defined, leading to growth arrest and cellular senescence, in part interacting with each other. Shortening of telomeres can be stopped by cell cycle arrest to protect the end of chromosomes of erosion. Besides telomeres the cell cycle inhibitors p16 (CDKN2A) and p21 (CDKN1A) have been found to play major roles in cellular senescence. While CDKN1A induction is directly related to telomere shortening via the p53 pathway, CDKN2A has been shown to induce cellular senescence independently of telomere attrition [9]. Functional kidney decline results in an accumulation of urea and other substances interfering with many cellular pathways including cellular senescence [10].

High-throughput Omics technologies coupled with bioinformatics analyses have paved the way for systematic studies of molecular mechanisms associated with age-related diseases. Blankenburg and colleagues for example recently consolidated over thirty age-related Omics datasets to construct a catalogue of common aging mechanisms with a particular focus on mechanisms and molecular features linking different age-related processes [11]. Data integration approaches also lead to the identification of novel age-related genes. Jiang and colleagues used RNA sequencing to screen for age-related genes being associated with IgA nephropathy to search for novel therapeutic targets [12]. Researchers have also started making use of available drug mechanism of action datasets as well as 3D drug models to identify compounds with potential beneficial effects on molecules and processes being deregulated in aging [13,14].

In this work we generated a comprehensive set of renal age-associated genes (RAAGs) via literature mining and Omics data analysis. This constructed set of RAAGs was mechanistically analysed on the level of molecular pathways. RAAGs being significantly associated with CKD progression on the transcriptional level served as input for in-silico compound screening. Compounds with a positive impact on aging and disease mechanisms by reversing RAAG expression changes were identified and are discussed in this work.

## 2 Material and Methods

### 2.1 Constructing the Renal Age-Associated Gene (RAAG) Set

We mined scientific literature for RAAGs using the following PubMed query: “*(aging OR ageing OR replicative senescence) AND (renal OR kidney)*” in February 2018. Human genes were extracted from the resulting set of publications using NCBI's gene2pubmed mapping file [15]. Gene-publication associations of all genes with at least two publications were manually curated to only include true positive age-related genes for further analysis.

We furthermore downloaded genes associated with the Gene Ontology (GO) term “aging” from the GO webpage at www.geneontology.org [16] as well as from the GenAge database [17]. We used data from the Human Protein Atlas on protein abundance and RNA expression in renal tissue to limit the gene sets extracted from GO and GenAge to those being expressed in kidney tissue [18]. We specifically focused on proteins with medium or high abundance in either glomerular or tubular tissue. We in addition determined the median RNA expression values for all three RNA datasets provided in the Human Protein Atlas of these proteins with medium or high abundance in glomerulus or tubules. Genes showing an RNA expression value above the calculated median RNA expression were also considered as relevant and included in the set of RAAGs. Another source for RAAGs was the Digital Aging Database [19]. RAAGs linked to kidney aging were downloaded using the respective search forms at the Digital Aging Database. The set of RAAGs extracted from scientific literature or age-related databases was complemented by including genes from transcriptomics studies. The gene sets published by Rodwell and colleagues as well as by Melk and colleagues were retrieved from the supplementary data files from the two published articles [20,21].

The rentrez R package (https://cran.r-project.org/web/packages/rentrez/index.html) was used to annotate transcripts in the supplementary table of Melk et al. via mapping the provided GenBank accession numbers to the respective NCBI UniGene cluster IDs and subsequently to the official Gene Symbols. The Ensembl Gene ID was used as common gene identifier and RAAGs were furthermore annotated with their official Gene Symbols.

### 2.2 Determining Protein Abundance in Renal Tissue

Protein abundance levels in renal tissue were retrieved from the kidney expression summary section of the Human Protein Atlas for all RAAGs [18]. Immunohistochemistry images have been manually annotated by two independent researchers and protein abundance levels are reported using one of the following four categories: “not detected”, “low”, “medium”, or “high”. The protein abundance levels are based on antibody staining intensity and fraction of stained cells. For kidney tissue, there is one protein abundance level for tubules and one for glomeruli provided in the Human Protein Atlas.

### 2.3 Investigating Associations with CKD Progression

The expression of RAAGs was evaluated in a transcriptomics data set consisting of 63 CKD patients [22]. Follow-up data were updated in Q2 2018 and patients were divided into a group with stable course of disease and a group with progressive course of disease. Progression was defined as either reaching end-stage renal disease or experiencing doubling of serum creatinine during the follow-up time with a minimum follow-up time of six months. Patients in the stable group did not develop ESRD nor doubling of serum creatinine. We further only kept patients with at least one year of follow-up in the stable group.

The institutional Review Board of the Medical University of Innsbruck has accredited the use of surplus material from routine kidney biopsies and anonymized patient data for research purposes.

Expression data are available in NCBI's Gene Expression Omnibus with the accession number GSE60861 (https://www.ncbi.nlm.nih.gov/geo/query/acc.cgi?acc=gse60861). The R limma package (https://bioconductor.org/packages/release/bioc/html/limma.html) was used for preprocessing and statistical analysis. Expression data were background corrected, quantile normalized, and probes with the same sequence were summarized. Differentially expressed RAAGs between the group of stable and progressive patients were identified using the Statistical Analysis of Microarrays (SAM) method setting the false discovery rate (FDR) to <2.5% with a fold-change cutoff of > |1.25|.

### 2.4 Analysing the Functional Context of RAAGs

Enriched molecular pathways were determined based on the set of RAAGs using the Database of Annotation, Visualization and Integrated Discovery (DAVID) v6.8 tool [23]. The Kyoto Encyclopedia of Gene and Genomes (KEGG) pathway set was used as underlying pathway resource. Enriched pathways with *p*-values <.05 after Bonferroni adjustment for multiple testing were considered as relevant. Disease-specific pathways such as “Type 2 diabetes mellitus” or “Pathways in cancer” were excluded from further analyses.

### 2.5 Identifying Compounds Reversing CKD Associated RAAGs

The library of integrated network-based cellular signatures (LINCS) L1000 data set was used to identify compounds reversing the set of RAAGs showing significant association with CKD outcome [24]. The LINCS L1000 data set holds over a million gene expression profiles for around 20,000 compounds tested in different concentrations and cell lines to study drug mechanism of action on a molecular level. We restricted our input signature for the *L1000 Characteristic Direction Signature Search Engine* (http://amp.pharm.mssm.edu/L1000CDS2/) to those RAAGs showing a concerted way of expression in renal aging and renal disease, i.e. being either up- or downregulated in both, renal aging and the group of progressive CKD patients. The 50 top-ranked compounds based on the drug score were further evaluated focusing in particular on the individual compound-gene combinations leading to high scores in the compound-RAAG signature interaction. The drug score is calculated based on the overlap of input RAAGs and the drug signature genes normalized to the effective input size defined as the number of intersecting genes between the input gene set and the set of L1000 genes.

### 2.6 Validating the Impact of Identified Compounds in Human Renal Proximal Tubular Cells

Proximal tubular human kidney cells (HK2) were purchased from American Type Culture Collection (CRL-2190, Wesel, Germany) and cultured in Keratinocyte-Serum Free Medium (KSFM) containing 10% fetal bovine serum (FBS), 5 ng/ml recombinant epidermal growth factor (rEGF), 0.05 mg/ml bovine pituitary extract (BPE), 100 U/ml penicillin and 100 μg/ml streptomycin. Cell culture supplies were purchased from ThermoFisher Scientific, Vienna, Austria. All cells were grown at 37 °C in a humidified atmosphere with 5% CO_2_. After growth to confluence, cells were pre-treated with 0.5 μM H_2_O_2_ for two hours to induce oxidative stress. Subsequently cells were stimulated with atorvastatin, captopril, rosiglitazone (10 μM each) or valsartan (1 μM). All chemicals used for stimulation experiments were purchased from Sigma-Aldrich, Vienna, Austria and solubilized in DMSO prior to use. Drug concentrations were chosen based on prior studies [[25], [26], [27], [28]]. After 24 h, RNA was isolated with RNeasy Mini Kit (Qiagen, Valencia, CA, USA) according to the manufacturer's protocol. RNA yield and quality were determined using a DS-11 FX+ spectrophotometer (DeNovix, Wilmington, DE, USA).

For qPCR, mRNA was reverse transcribed into cDNA with the High Capacity cDNA reverse Transcription kit. Samples were analysed with the following TaqMan® Gene Expression Assays: C3 (Hs00163811_m1), GAPDH (Hs99999905_m1), EGF (Hs01099999_m1), CD52 (Hs00174349_m1), CFB (Hs00156060_m1), LTF (Hs00914334_m1), MMP7 (Hs01042796_m1), TNFRSF11B (Hs00900358_m1). All materials for qPCR were purchased from ThermoFisher Scientific, Vienna, Austria.

Reactions were prepared in duplicate for each sample (technical replicates) and analysed on the 7500 Fast Real-Time PCR System (Applied Biosystems) under the following conditions: 50 °C for 2 min, 95 °C for 10 min followed by 40 cycles of 95 °C for 15 s and 60 °C for 1 min. Three biological replicates per condition were performed. The 2^-ddCt^ Method was used to calculate fold change values with respect to untreated control cells using GAPDH as housekeeper transcript [29]. Unpaired *t*-test assuming equal group variances was used to compare fold change values of H_2_O_2_ treated samples with H_2_O_2_ and drug-treated samples.

## 3 Results

### 3.1 The Renal Age-Associated Gene (RAAG) Set

**Fig. 1:**
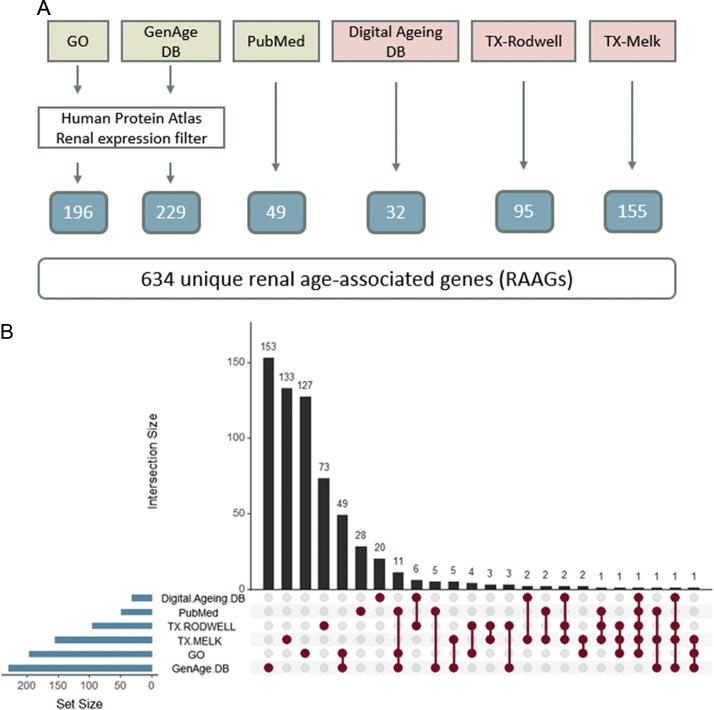
Generation of the renal age-associated gene (RAAG) set. Numbers of RAAGs extracted from the six different sources contributing to the final set of 634 unique RAAGs are provided in Fig. 1A. Transcriptomics-based data sets are highlighted in red with literature-based data sets shown in green. The overlap of individual RAAGs between the six different data sources is displayed in Fig. 1B.

Six different data sources were used to generate the RAAG signature consisting of 634 unique genes (Fig. 1 and Table 1).

**Table 1:** Listing of renal age-associated datasets.

Dataset acronym	Dataset description	# Genes	Ref
PubMed	Human genes were extracted from the gene2pubmed mapping file for publications found with the query: (aging OR ageing OR replicative senescence) AND (renal OR kidney)	49	[15]
GO	Genes associated with the GO term “aging” were retrieved and filtered for those being expressed in either tubules or glomeruli based on data from the Human Protein Atlas.	196	[16]
GenAge DB	Human genes were extracted from the GenAge DB and filtered for those being expressed in either tubules or glomeruli based on data from the Human Protein Atlas.	229	[17]
Digital Ageing DB	Human genes associated with renal aging were retrieved from the Digital Ageing DB.	32	[19]
TX-RODWELL	Genes associated with renal aging in the published transcriptomics dataset by Rodwell et al. were extracted from the supplementary datasets of the publication.	95	[20]
TX-MELK	Genes associated with renal aging in the published transcriptomics dataset by Melk et al. were extracted from the supplementary datasets of the publication.	155	[21]

Overview and brief description of datasets used for generating the RAAG signature in the present study.

Information from three sources, namely PubMed, GO and GenAge DB, was mainly based on literature information whereas information from the other three sources, namely the Digital Aging DB, TX-Rodwell, and TX-Melk, was based on high-throughput transcriptomics experiments. The largest contributions to the set of RAAGs were from the GenAge DB (229) and from the GO dataset (196), even after filtering for genes being expressed in kidney tissue based on information obtained from the Human Protein Atlas, as these sources are not restricted to and therefore not specific for renal aging per se. The overlap between these two literature-based sources was significant (*p*-value <.0001, chi-square test) with 49 genes being reported in both data sets. 49 RAAGs were extracted from resulting publications using the PubMed query “*(aging OR ageing OR replicative senescence) AND (renal OR kidney)*” after manual curation of human gene-to-pubmed entries. The transcriptomics datasets TX-Melk, TX-Rodwell, as well as the Digital Aging DB contributed 155, 95, and 32 RAAGs respectively.

Matrix metallopeptidase 7 (MMP7) showed up in five of the six data sets with its expression going up with increasing age. Next in the list was epidermal growth factor (EGF), which was found in four data sets, with its expression going down with increasing age. A number of genes was found in three data sets, among them the cyclin dependent kinase inhibitors 1A and 2A (CDKN1A and CDKN2A), klotho (KL), the mechanistic target of rapamycin kinase (MTOR), fibronectin 1 (FN1), or apolipoprotein E (APOE). The full list of RAAGs is available in Supplementary Table S1 along with information on the individual sources.

### 3.2 Renal Specific Protein Abundance

**Fig. 2:**
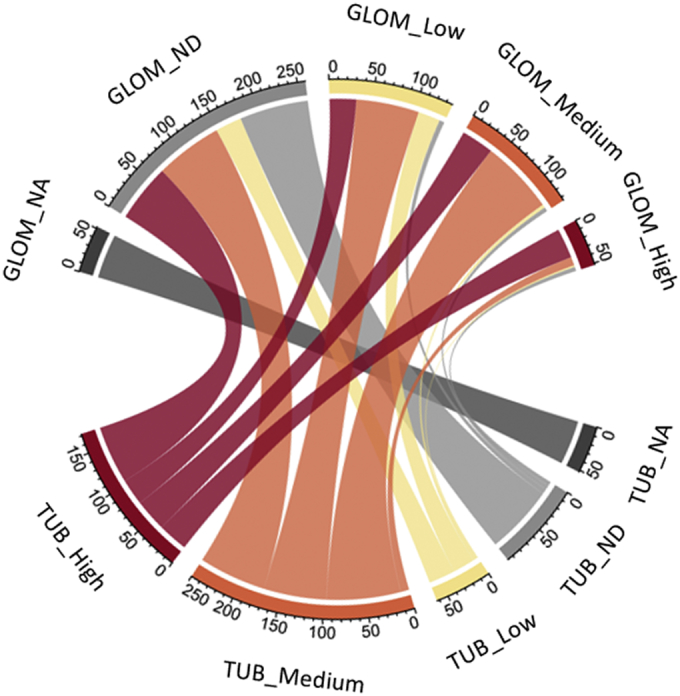
Renal age-associated gene (RAAG) protein abundance in renal tissue. Overview of protein abundance in renal tubules and glomeruli for the 634 RAAGs based on data from the Human Protein Atlas. The number of RAAGs in each of the different categories is indicated by segment sizes of the outer ring. Color-coding of the outer ring indicates high protein abundance (red), medium protein abundance (orange), or low protein abundance (yellow) in tubular and glomerular tissue respectively. The fraction of RAAGs being not detectable (ND) on the protein level are given in light grey. No immunohistochemistry information was available for 52 RAAGs in the Human Protein Atlas (GLOM_NA and TUB_NA), displayed in dark grey. Connecting line segments are color coded based on tubular protein abundance levels.

We evaluated RAAG expression in renal tissue using information from the Human Protein Atlas on protein abundance levels in glomerular and tubular compartments. Information was available for 582 of the 634 RAAGs. 485 RAAGs showed at least low levels of protein abundance in tubules, while at least low protein abundance in the glomerular compartment was observed in 323 RAAGs. Average protein abundance was higher in tubules as compared with glomeruli with around 250 RAAGs being not at all expressed in glomeruli. Fig. 2 displays protein expression of RAAGs in tubular and glomerular compartment based on data from the Human Protein Atlas.

### 3.3 Association with CKD Progression

We evaluated RAAG expression in renal tissue in a set of 63 CKD patients with available clinical follow-up data. Mean follow-up time of the whole cohort was 7.01 years and key clinical parameters are listed in Table 2. The percentage of male patients was higher in the progressive group (*p*-value = .0142, chi-square test) and patients were significantly older (p-value = .00057, *t*-test) as compared with the patients in the stable group. No significant difference between treatment was observed (p-value = .3338, chi-square test).

**Table 2:** Patient characteristics of the CKD cohort.

	Cohort (*N* = 63)	Progressive (N = 24)	Stable (*N* = 39)	p-value
Age [years]				
Min	17.06	20.00	17.06	
Max	74.00	74.00	73.85	
Mean (sd)	47.22 ± 17.49	56.19 ± 13.90	41.70 ± 17.32	0.00057
Creatinine [mg/dL]				
Min	0.44	0.44	0.49	
Max	6.21	6.21	4.18	
Mean (sd)	1.55 ± 0.95	1.96 ± 1.13	1.31 ± 0.73	0.01624
eGFR [ml/min/1.73 m^2^]				
Min	9.46	9.46	16.24	
Max	153.68	153.68	150.58	
Mean (sd)	63.28 ± 35.20	49.54 ± 32.74	71.73 ± 34.36	0.01335
UPCR [g/g]				
Min	0.22	0.72	0.22	
Max	12.77	11.23	12.77	
Mean (sd)	4.36 ± 3.37	4.22 ± 2.80	4.45 ± 3.71	0.78860
Follow-up time [years]				
Min	0.52	0.52	1.22	
Max	17.08	11.34	17.08	
Mean (sd)	7.01 ± 4.30	4.7 ± 3.24	8.44 ± 4.28	0.00024
Gender				
Female	27 (43%)	5 (21%)	22 (56%)	
Male	36 (57%)	19 (79%)	17 (44%)	0.01418
Medication at baseline [n (%)]				
RAAS blockade	29 (46)	13 (54)	16 (41)	
Antidiabetics	5 (8)	3 (13)	2 (5)	
Statins	13 (21)	6 (25)	7 (18)	
Steroids	12 (19)	3 (13)	9 (23)	
NSAIDs	2 (3)	0 (0)	2 (5)	
NA	10 (16)	6 (25)	4 (10)	0.33380
Histological diagnoses [n (%)]				
DN	5 (8)	5 (21)	0 (0)	
FSGS	7 (11)	3 (13)	4 (10)	
GN	1 (2)	1 (4)	0 (0)	
HTN	7 (11)	4 (17)	3 (8)	
IGAN	4 (6)	3 (13)	1 (3)	
LN	11 (17)	1 (4)	10 (26)	
MCD	10 (16)	0 (0)	10 (26)	
MN	10 (16)	4 (17)	6 (15)	
MPGN	2 (3)	2 (8)	0 (0)	
VASC	5 (8)	1 (4)	4 (10)	
Other	1 (2)	0 (0)	1 (3)	0.56460

Clinical parameters are given for the CKD cohort [*n* = 63] as well as for the subsets of patients with a stable [*n* = 39] and progressive [*n* = 24] course of disease. eGFR = estimated glomerular filtration rate; UPCR = urine protein to creatinine ratio; RAAS = renin angiotensin aldosterone system; NSAIDs = non-steroidal anti-inflammatory drugs; DN = diabetic nephropathy; FSGS = focal segmental glomerulosclerosis; GN = glomerulonephritis; HTN = hypertensive nephropathy; IGAN = IgA glomerulonephritis; LN = lupus nephritis; MCD = minimal change disease; MN = membranous nephropathy; MPGN = membranoproliferative glomerulonephritis; VASC = vasculitis.

31 RAAGs were identified as differentially expressed in the group of 24 progressive CKD Patients as Compared with the Group of 39 CKD Patients Showing a Stable Disease course (Table 3). Patient Core Clinical Parameters and Information on Assignment to the Progressive and Stable Group Can Be Found in Supplementary Table S2

The gene set being significantly deregulated in progressive CKD was enriched in RAAGs from the TX-RODWELL (p-value <.00001) and the Digital Aging DB (p-value = .0004) datasets, both transcriptomics-based datasets. In contrast a significantly lower number of RAAGs than expected was present from the GenAge DB (p-value = .0456) based on a chi-square test.

**Table 3:** RAAGs associated with CKD progression.

Symbol	Gene name	Aging evidence	Protein abundance in glomeruli	Protein abundance in tubules	Fold-change CKD	p-Value	FDR (%)
C3	Complement C3	TX-RODWELL	Not detected	Not detected	2.69	2.41E-05	< 1%
LTF	Lactotransferrin	Digital Aging DB; TX-RODWELL	Not detected	Not detected	2.61	3.47E-05	< 1%
IL7R	Interleukin 7 receptor	GenAge DB	Not detected	Medium	2.22	3.09E-04	< 1%
NNMT	Nicotinamide *N*-Methyltransferase	Digital Aging DB; TX-RODWELL	Not detected	High	2.19	2.39E-04	< 1%
CD52	CD52 Molecule	TX-MELK	NA	NA	2.10	3.48E-04	< 1%
UBD	Ubiquitin D	TX-RODWELL	NA	NA	1.82	1.43E-03	< 1%
APBB1IP	Amyloid beta precursor Protein binding family B Member 1 interacting protein	Digital Aging DB	Not detected	Not detected	1.73	1.79E-05	< 1%
CFB	Complement factor B	TX-RODWELL	Not detected	Not detected	1.73	5.96E-03	< 1%
MMP7	Matrix metallopeptidase 7	PubMed; GO; Digital Aging DB; TX-RODWELL; TX-MELK	Not detected	Low	1.63	2.22E-03	< 1%
VCAN	Versican	TX-MELK	Low	Low	1.57	2.91E-04	< 1%
CLDN1	Claudin 1	GO	Not detected	Medium	1.57	3.77E-05	< 1%
ITGB2	Integrin subunit beta 2	GO	Not detected	Not detected	1.57	1.37E-02	< 3%
MICALL2	MICAL-like 2	TX-RODWELL	Not detected	Low	1.56	3.37E-05	< 1%
INPP5D	Inositol polyphosphate-5-phosphatase D	GO	Not detected	High	1.54	1.06E-04	< 1%
CLU	Clusterin	GenAge DB	Not detected	Not detected	1.49	8.61E-03	< 2%
ITPR3	Inositol 1,4,5-trisphosphate receptor type 3	TX-RODWELL	Not detected	Low	1.46	6.41E-06	< 1%
TMPRSS4	Transmembrane serine protease 4	TX-RODWELL	Low	Low	1.41	5.11E-04	< 1%
WFDC2	WAP four-disulfide core domain 2	TX-RODWELL	Not detected	Low	1.40	1.23E-02	< 2%
CDH6	Cadherin 6	TX-RODWELL	Low	High	1.40	5.76E-03	< 1%
TSPAN1	Tetraspanin 1	TX-RODWELL	Not detected	High	1.38	1.18E-02	< 2%
ANXA1	Annexin A1	TX-RODWELL	Medium	Not detected	1.36	4.01E-03	< 1%
GLS	Glutaminase	TX-MELK	Not detected	High	1.32	1.78E-03	< 1%
TNFRSF11B	TNF receptor superfamily member 11b	TX-RODWELL	Not detected	High	1.32	1.57E-03	< 1%
CXCL6	C-X-C motif chemokine ligand 6	TX-RODWELL	NA	NA	1.28	1.01E-03	< 1%
TPBG	Trophoblast glycoprotein	TX-RODWELL	Medium	High	1.27	6.49E-05	< 1%
NOS3	Nitric oxide synthase 3	PubMed	Low	Not detected	−1.27	3.32E-04	< 1%
CGNL1	CINGULIN like 1	TX-MELK	Medium	High	−1.27	1.30E-03	< 2%
STARD8	StAR related lipid transfer domain containing 8	Digital Aging DB	Low	High	−1.34	2.54E-04	< 1%
CDKN2A	CYCLIN dependent kinase inhibitor 2A	PubMed; GO; GenAge DB	Medium	Medium	−1.38	4.08E-04	< 1%
LPL	Lipoprotein lipase	Digital Aging DB; TX-RODWELL	NA	NA	−1.79	1.34E-03	< 1%
EGF	Epidermal growth factor	GenAge DB; Digital Aging DB; TX-RODWELL; TX-MELK	NA	NA	−2.06	2.11E-04	< 1%

Listing of RAAGs being significantly associated with CKD progression based on the comparison of 24 progressive and 39 stable CKD patients. Next to the Gene Symbol and the gene name, we provide information on the aging data sets as well as protein abundance levels in tubules and glomeruli. In addition fold-change values in progressive CKD along with *p*-values and FDR (%) are provided. RAAGs are sorted based on fold-change values in descending order.

**Fig. 3:**
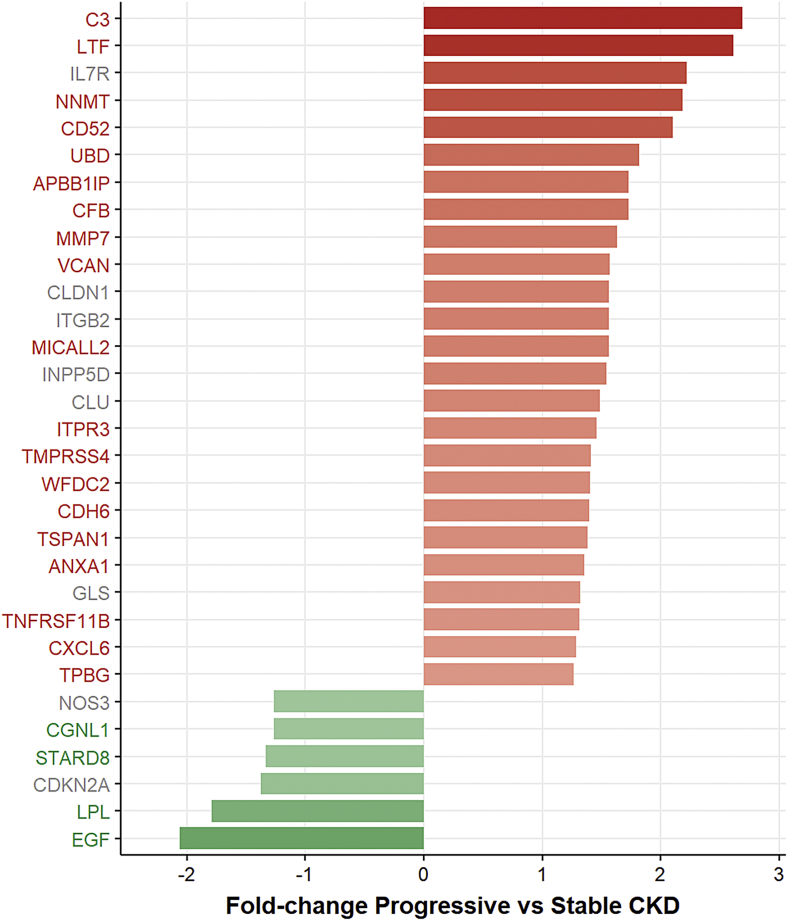
Renal age-associated gene (RAAGs) associated with CKD progression. Barplot of RAAG fold changes when comparing expression data from progressive CKD patients with CKD patients showing a stable course of disease. Upregulated and downregulated genes in progressive CKD patients are indicated by red and green bars respectively. Genes showing concordant regulation of expression in CKD and renal aging are indicated by colored labels. Grey labels are used for genes showing discordant regulation of expression in CKD and renal aging.

25 of the 31 differentially expressed RAAGs were upregulated and six RAAGs were downregulated in the group of progressive patients as compared with the group of stable CKD patients. 19 of the 25 upregulated RAAGs showed a concordant pattern of expression with aging, i.e. being significantly upregulated in progressive CKD patients and being also reported as significantly upregulated with increasing age. Four of the six downregulated RAAGs on the other hand showed a concordant pattern in the opposite direction, i.e. being downregulated in progressive CKD patients and being reported to show decreased expression with increasing age. The RAAGs with concordant expression patterns in CKD and aging are highlighted in Fig. 3 with colored labels.

EGF was downregulated 2.06-fold in progressive CKD patients. C3 and lactotransferrin (LTF) on the other hand showed the largest upregulations in progressive CKD patients as compared with CKD patients showing a stable disease course with fold-change values of 2.69 and 2.61, respectively.

### 3.4 Enriched Molecular Pathways and Functional Context

**Fig. 4:**
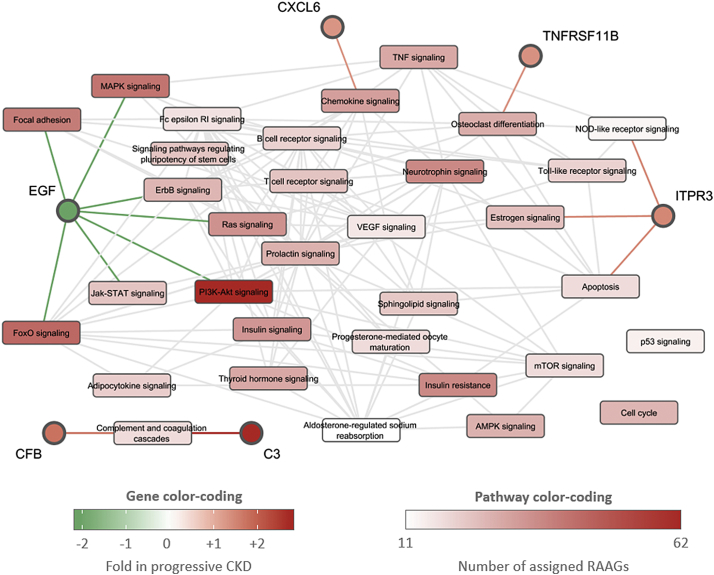
Pathway map based on renal age-associated gene (RAAGs). Enriched molecular pathways based on the set of 634 RAAGs are displayed. Pathway color-coding indicates number of assigned RAAGs. Inter-pathway links indicate pathways sharing a common set of genes. RAAGs showing concordant regulation in CKD progression that could be assigned to at least one of the enriched pathways are displayed in addition with the color-coding indicating fold-changes in the CKD transcriptomics dataset.

33 enriched molecular KEGG pathways were identified using the signature of 634 RAAGs as input for the DAVID tool with a Bonferroni corrected *p*-value of <0.05. Among them, we identified well-known age-related signaling cascades such as cell cycle, mTOR signaling, P53 signaling, insulin signaling, or focal adhesion. A representation of enriched molecular pathways is displayed in Fig. 4. Next to the enriched pathways, those genes showing concordant expression in CKD and renal aging that could be assigned to at least one of the enriched molecular pathways, are displayed.

EGF is linked to the largest number of the 33 significantly enriched molecular pathways based on the signature of 634 RAAGs. Next to the ErbB signaling pathway itself, also PI3K-Akt signaling and Jak-STAT signaling play a crucial role, which has also been shown for focal adhesion, prominently represented by MMP7, FOXO, or RAS.

A number of other RAAGs found to be significantly associated with CKD progression could be assigned to the broader context of cell adhesion and extracellular matrix remodeling including VCAN, MMP7, ITGB2, CLDN1, TSPAN1, TPBG, or CGNL1 based on information from GO terms and scientific literature. RAAGs involved in immune response and inflammation furthermore included complement C3 (C3), IL7R, CD52, UBD, CLU, ANXA1, and CXCL6.

### 3.5 Compounds Reversing the RAAG/CKD Profile

**Fig. 5:**
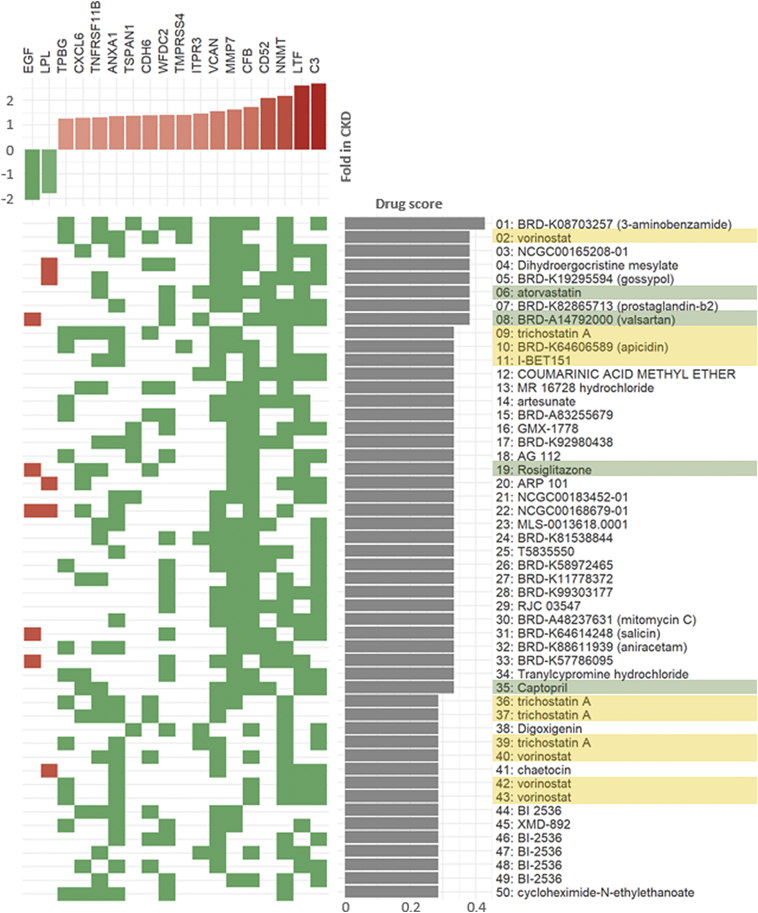
Heatmap of compound–renal age-associated gene (RAAG) interactions. The impact of the 50 top-ranked compounds on RAAG expression is indicated in this heatmap. Compounds in part reversing the RAAG/CKD signature are ranked from top to bottom on the right hand side. Affected RAAGs are ranked from left to right in the top panel. Red squares in the heatmap indicate genes being upregulated by a specific drug, whereas green squares indicate genes being downregulated by a particular drug. Compounds in use in the context of diabetes and kidney disease are highlighted in green. Compounds targeting epigenetic mechanisms and in particular histone deacetylase inhibitors are highlighted in yellow. Compounds showing up multiple times in the list have been found to be of relevance in different cell lines and/or different concentrations. Details on experimental conditions in the drug profiling experiments are provided in Supplementary Table S3.

The set of 23 RAAGs showing concordant expression patterns in CKD and renal aging was used as input for in-silico drug screening using the *L1000 Characteristic Direction Signature Search Engine.* The 50 top-ranked compounds reversing the input RAAG signature were evaluated in detail with respect to the impact on individual RAAG expression levels. The heatmap in Fig. 5 displays those 18 RAAGs as column headers being affected by at least one of the top-ranked compounds. The compounds are sorted based on the resulting drug score from the *L1000 Search* from top to bottom.

Among the set of identified compounds were four drugs being already used in the clinical setting in the context of diabetes and kidney disease, namely atorvastatin, valsartan, rosiglitazone, and captopril. Next to these four compounds there was a set of compounds from the class of histone deacetylase (HDAC) inhibitors in the list such as vorinostat, trichostatin A, apicidin, or I-BET151. The majority of the remaining identified compounds were investigational drugs. A detailed list of the top-ranked compounds with information on the underlying cell-line, dose, and time is available in Supplementary Table S3.

### 3.6 Impact of Identified Compounds on CKD Associated RAAGs in Human Renal Proximal Tubular Cells

To validate the findings from our in-silico prediction workflow, we tested the impact of four compounds (atorvastatin, valsartan, rosiglitazone, and captopril) on gene expression levels in human renal proximal tubular cells of selected RAAGs that were also associated with CKD progression. We selected RAAGs for which either literature information was available on the impact for at least one of the four drugs or that should be affected by at least three of the four drugs based on the in-silico predictions. The selected genes were EGF, TNF receptor superfamily member 11b (TNFRSF11B), MMP7, complement factor B (CFB), CD52, LTF, and C3. No valid relative concentrations could be determined for CD52 via qPCR and CD52 was therefore excluded from further analyses.

**Fig. 6:**
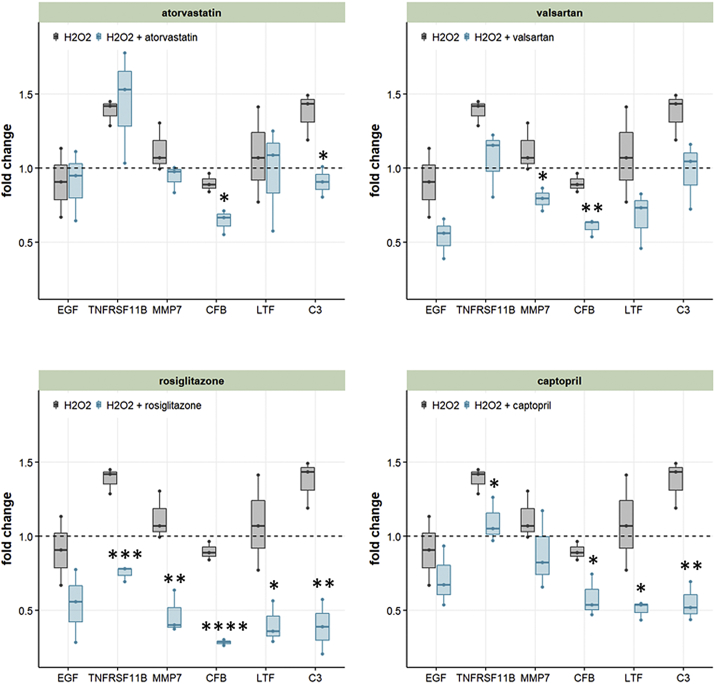
Drug impact on renal age-associated gene (RAAG) expression in HK2 cells. The impact of atorvastatin, valsartan, rosiglitazone, and captopril on RAAG gene expression in HK2 cells is displayed. Data represent expression fold changes as compared with untreated HK2 controls after normalization to expression levels of the housekeeping gene GAPDH. T-test was used to assess significance between H_2_O_2_ stimulated cells and those treated with the respective drugs on top of H_2_O_2_ stimulation. p-value coding: * < 0.05; ** < 0.01; *** < 0.001; **** < 0.0001.

Expression of C3 and TNFRSF11B was upregulated 1.37 and 1.38-fold by H_2_O_2_ stimulus as compared with untreated HK2 controls. Rosiglitazone and captopril had the strongest impact on gene expression levels significantly counterbalancing expression levels of five and four of the selected genes, respectively (Fig. 6). Valsartan and atorvastatin both significantly reduced expression of two RAAGs. In particular the two RAAGs linked to the complement and coagulation cascade C3 and CFB were affected by the selected compounds, with CFB being significantly downregulated by all four compounds and C3 being significantly downregulated by three of the four tested compounds.

## 4 Discussion

In this study, we established and applied an in-silico analysis approach to identify compounds reversing a set of RAAGs also being significantly associated with CKD progression. We (i) consolidated a set of 634 RAAGs based on six different data sets, (ii) determined protein abundance in renal compartments, (iii) analysed enriched molecular pathways based on the RAAG signature, (iv) generated a set of RAAGs being also significantly associated with CKD progression in an independent transcriptomics dataset, (v) identified a set of compounds with the potential of reversing the expression signature of renal age-associated CKD prognosis genes, and (vi) validated the impact of selected drugs on RAAG expression in a cell culture model system of renal aging.

A number of studies on renal aging is available in scientific literature, but to the best of our knowledge no data driven meta-analysis has been conducted so far. With three literature-based data sets and three gene expression data sets, we generated a unique set of 634 genes being associated with renal aging. Interestingly the overlap between genes from the six different data sets was sparse with the largest overlap found between the two literature-based data sets from GO and the GenAge DB. Over one third of RAAGs from the Digital Aging DB however were also part of one of the two transcriptomics datasets TX-RODWELL or TX-MELK. The remaining datasets seemed to provide complementary findings.

Among the set of 33 identified enriched molecular pathways based on the signature of 634 RAAGs are key age-related mechanisms such as the mTOR pathway, the p53 signaling cascade, or cell cycle regulation but also mechanisms like focal adhesion or insulin resistance. mTOR signaling modulates aging and age-related diseases [30] while insulin resistance amplifies chronic inflammation leading to accelerated aging [31]. Unresolved inflammatory processes in renal transplants trigger delayed graft function involving aging pathways suggesting a dominant role of inflammation in the process of renal aging [32]. Cell cycle regulation has been discussed widely as major factor in the process of cellular aging also in the field of nephrology [33,34], whereas the p53 family is involved in DNA repair interacting with cellular aging [35].

The hunt for compounds with a positive impact on molecular processes of aging is an intriguing area of research. A couple of substances have already been identified such as compounds reducing the effect of reactive oxygen species or compounds targeting the mTOR complex with different hypotheses to expand life span including specific translation of mRNAs, improving stem cell maintenance or anti-inflammatory mechanisms [30,36,37]. Using the set of 23 RAAGs showing concordant regulation in aging and CKD progression we identified compounds with the potential to reverse the RAAG/CKD expression signature. Drugs used to treat diabetes, hypertension, or hypercholesteremia (rosiglitazone, captopril, valsartan, and atorvastatin) showed up in this list, all directly affecting renal function [38,39].

We investigated the impact of these four drugs on RAAG expression levels in a cell culture model system of aging, namely treatment of renal proximal tubular HK2 cells with H_2_O_2_ [40]. All four investigated drugs showed significant beneficial impact on RAAG gene expression with the antidiabetic drug rosiglitazone having the strongest impact targeting five of the six selected genes. There is also evidence for the impact of rosiglitazone on LTF, MMP7, and TNFRSF11B supporting our findings [41], [42]. The two complement and coagulation factors C3 and CFB were affected by all compounds in our in-vitro model system. Activation of the alternative pathway of the complement system has been discussed as a link between obesity and metabolic disorders. Rosiglitazone leads to a significant reduction of CFB, which is positively correlated to a number of obesity associated parameters [43]. Statins also showed beneficial impact on the level of components of the complement and coagulation cascade in a cohort of patients with systemic sclerosis [44].

Next to these in-vitro tested compounds, epigenetic regulators such as the HDAC inhibitors vorinostat, trichostatin A, or apicidin along with the bromodomain and extra-terminal motif (BET) inhibitor I-BET151 scored high in our in-silico ranking. Histone deacetylases are enzymes regulating the modification of chromatin arrangement, transcriptional activity, and are involved in enabling epigenetic integrity. Renoprotective effects of HDAC inhibitors have been described in combination with inhibitors of the RAAS [45]. This group of compounds was also found to have a positive impact in the context of aging in the human brain [14]. Epigenetic regulation as therapeutic option in kidney disease however seems to be a two-edged sword where the therapeutic benefit has to be weighed against the nephrotoxic potential as reviewed recently [46].

We compared our results to a list of potential anti-aging compounds, which was consolidated by Dönertaş and colleagues based on the results of twelve different studies [47]. The two HDAC inhibitors vorinostat and trichostatin A were part of the consolidated list being among the few compounds with at least three supporting studies. The ACE inhibitors captopril and enalapril were also listed next to pravastatin, a member of the drug class of statins like atorvastatin, which was identified in our study. The relevance to human aging and disease however needs to be established, as the evidence for the link to aging for the ACE inhibitors as well as pravastatin was based on studies in rotifers [48].

Those RAAGs being associated with CKD progression and being affected by one of the compounds in clinical use for diabetes and/or kidney disease might help in this task and might all be considered as potential predictive marker candidates for the respective drugs. CKD patients with elevated levels of certain RAAGs might for example have a larger benefit from therapeutic intervention than patients with normal RAAG expression levels. Assessment of these proteins as predictive markers was however beyond the scope of the present work but will be the focus of future follow-up studies.

Findings of the present study are also of relevance to all future nephrological biomarker studies. As soon as one of the RAAGs is investigated as biomarker candidate in the context of kidney disease, adjustment for age is probably even more important in order to draw correct conclusions from the respective biomarker study. It might turn out, that the value of the biomarker is merely due to its association with chronological age, which still is the strongest single predictor of overall mortality [49].

This exploratory study has its limitations. Despite the fact that we applied quite stringent criteria on the underlying age-related data sets there is a chance that a certain amount of genes in the RAAG set is of minor relevance to renal aging and has therefore to be considered as false positives. In particular the initial gene sets extracted from GO and the GenAgeDB needed another curation step, as neither of the two sources specifically focused on renal aging alone. To reduce the number of false positives we therefore applied a filter using data from the Human Protein Atlas on gene and protein abundance values in renal tissue. After the pathway enrichment analysis, we however felt very positive about the generated set of RAAGs, as a number of aging and kidney disease related molecular pathways showed up on top of the list.

## 5 Conclusions

In summary, we consolidated a set of RAAGs based on six independent data sources. RAAGs being significantly associated with CKD progression in an independent transcriptomics dataset were used to screen for compounds reversing the RAAG/CKD signature. Atorvastatin, valsartan, rosiglitazone, and captopril – all drugs being widely used in clinical routine to treat hypertension and diabetes, two conditions causing up to 50% of all cases of end-stage renal disease – were among the top-ranked compounds. In particular rosiglitazone and captopril had a significant beneficial impact on RAAG expression levels in human renal proximal tubular cells. In addition, compounds targeting epigenetic regulatory mechanisms were identified impacting renal age-related mechanisms.

The following are the supplementary data related to this article.

Supplementary Table S1 Complete renal age-associated gene (RAAG) set Supplementary Table 1 holds the complete list of 634 RAAGs along with information on the underlying data sources. The Gene Symbol as well as the Ensembl GeneID is provided as gene identifiers. Supplementary Table S1

Supplementary Table S2 CKD patient cohort The set of 63 CKD patients used in this study in order to identify RAAGs being associated with CKD progression is provided in supplementary table 2. The provided patient IDs can be used in order to retrieve the respective gene expression datasets from GEO with the accession number GSE60861. Supplementary Table S2

Supplementary Table S3 Compounds reversing the renal age-associated gene (RAAG)/CKD signature The detailed listing of the 50 top-ranked compounds reversing the RAAG/CKD signature is available in supplementary table 3 with information on the calculated drug scores as well as information on used cell-lines, doses and time points of the underlying compound profiling data. Supplementary Table S3

## Author Contributions

CK and PP designed and coordinated the study. CK and PP led the analysis with JL and MR supporting data consolidation and analysis. MR and JK consolidated clinical data of the CKD patient cohort used within this study. JL performed cell culture experiments. CK and PP drafted the first version of the manuscript. All authors contributed to results interpretation and manuscript writing. All authors read and approved the final manuscript.

## Declarations of Competing Interests

All authors have declared that no conflict of interest exists.
